# Effect of Platelet-Rich Plasma (PRP) versus Autologous Whole Blood on Pain and Function Improvement in Tennis Elbow: A Randomized Clinical Trial

**DOI:** 10.1155/2014/191525

**Published:** 2014-01-20

**Authors:** Seyed Ahmad Raeissadat, Leyla Sedighipour, Seyed Mansoor Rayegani, Mohammad Hasan Bahrami, Masume Bayat, Rosa Rahimi

**Affiliations:** ^1^Department of Physical Medicine and Rehabilitation, Shahid Modarres Hospital, Faculty of Medicine, Shahid Beheshti University of Medical Sciences, Tehran 1998734383, Iran; ^2^Department of Physical Medicine and Rehabilitation, Shohadaye Tajrish Hospital, Faculty of Medicine, Shahid Beheshti University of Medical Sciences, Tehran 1989934148, Iran

## Abstract

*Background.* Autologous whole blood and platelet-rich plasma (PRP) have been both suggested to treat chronic tennis elbow. The aim of the present study was to compare the effects of PRP versus autologous whole blood local injection in chronic tennis elbow. *Methods.* Forty patients with tennis elbow were randomly divided into 2 groups. Group 1 was treated with a single injection of 2 mL of autologous PRP and group 2 with 2 mL of autologous blood. Tennis elbow strap, stretching, and strengthening exercises were administered for both groups during a 2-month followup. Pain and functional improvements were assessed using visual analog scale (VAS), modified Mayo Clinic performance index for the elbow, and pressure pain threshold (PPT) at 0, 4, and 8 weeks. *Results.* All pain and functional variables including VAS, PPT, and Mayo scores improved significantly in both groups 4 weeks after injection. No statistically significant difference was noted between groups regarding pain scores in 4-week follow-up examination (*P* > 0.05). At 8-week reevaluations, VAS and Mayo scores improved only in PRP group (*P* < 0.05). *Conclusion.* PRP and autologous whole blood injections are both effective to treat chronic lateral epicondylitis. PRP might be slightly superior in 8-week followup. However, further studies are suggested to get definite conclusion.

## 1. Background

Lateral epicondylitis known as tennis elbow is a repetitive strain injury caused by repetitive overuse of the extensor muscles of the wrist. It is the most frequent type of myotendinosis occurring more specifically at the common extensor tendon that originates from the lateral epicondyle [1, 2]. The frequency of lateral epicondylitis is reported between 1 to 3% among normal nonathlete population [3].

Epicondylitis was initially believed to be an inflammatory process but in 1979, it was described as the disorganization of normal collagen architecture by invading fibroblasts in association with an immature vascular reparative response, which termed “angiofibroblastic hyperplasia” [1, 2]. It causes pain and functional impairment in daily activities [2, 3]. The treatment of this condition includes conservative therapy and surgical interventions [3, 4]. The effectiveness of oral nonsteroidal anti-inflammatory agents, topical and injectable medications including corticosteroids and botulinum toxins, splinting, physical therapy, and iontophoresis has been evaluated in many studies [4]. However, these traditional therapies do not alter the tendon's inherent poor healing properties secondary to poor vascularization [5, 6]. Given the inherent nature of the tendon, new treatment options including platelets rich plasma (PRP), autologous blood, and prolotherapy are aimed at inducing inflammation rather than suppressing it [7–9]. PRP is quite a new treatment used for chronic tendinitis [4]. platelet rich plasma is defined as a volume of the plasma fraction of autologous blood having a platelet concentration above baseline [6]. Both PRP and autologus blood contain platelets, and these platelets have strong growth factors and granules that have critical role in the healing process of chronic injuries [7, 8]. Due to higher concentration of platelets in PRP than whole blood, it was shown to have greater effect in the repair process in treatment of chronic nonhealing tendinopathies including tennis elbow [4, 8, 9]. Therapeutic PRP should have a platelet concentration 4 to 6 times greater than that of whole blood (200000/mm^3^). The concentrations less than or greater than this amount may be ineffective or inversely lead to suppression of the healing process [4, 6, 7]. Some studies have shown that local injection of autologus whole blood has greater therapeutic effect than steroid injection in treating tennis elbow [5, 10, 11]; also there are studies showing the greater efficacy of local autologous PRP than corticosteroids in treating this disorder [4, 8]. However, only a few studies have been conducted to compare the efficacy of these two treatments. A comparative study of these 2 treatments was conducted by Thanasas et al. in 2011 in an effort to investigate the possible advantages of PRP versus autologous whole blood for the treatment of chronic tennis elbow. Six weeks after the therapy, PRP treatment seemed to be more effective than autologous blood in reducing pain [12]. However, this study and most of the other similar studies lacked objective evaluations of symptom improvements after whole blood or PRP injection.

Considering the high cost of autologous PRP therapy and lack of a study comparing autologous whole blood versus PRP injection objectively, this study was designed to evaluate the efficacy of autologous whole blood injection as a less costly treatment versus PRP in patients suffering from chronic lateral epicondylitis.

## 2. Methods

### 2.1. Patients and Setting

All patients with clinical signs and symptoms of chronic lateral epicondylitis during May 2011–May 2012 referring to the physical medicine and rehabilitation clinic of Shahid Modarres Hospital which is a general educational hospital were evaluated to enter this randomized, single blind study.

### 2.2. Inclusion Criteria

Criteria for inclusion in the study were chronic clinically diagnosed lateral epicondylitis (based on symptoms, site of tenderness, and pain elicited with resisted active extension of the wrist in pronation and elbow extension); with duration of symptoms more than 3 months and pain severity with minimum score of 5 (based on 10 scale Visual Analogue Score (VAS)).

### 2.3. Exclusion Criteria

Patients were excluded if they were pregnant, older than 75 years, had history of trauma, any platelet dysfunction syndrome (Critical thrombocytopenia), any other coagulopathies (such as hypofibrinogenemia), local infection at the site of the procedure, any recent febrile or infectious disease, consistent use of NSAIDs within 48 hours before procedure, recent use of corticosteroids during last 2 weeks, a history of local injection of any medications (steroid, whole blood, PRP, or dry needling) into the site of lateral epicondyle, hemoglobin <10 gr/dL, plasma platelets count <100000/mm^3^, history of any malignancy (including hematologic and non hematologic malignancies), carpal tunnel syndrome, cervical radiculopathy or peripheral radial nerve injury, systemic illnesses including ischemic heart disease, diabetes, rheumatoid arthritis, hepatitis, any bony malformations, bony or articular lesions at elbow (diagnosed by radiographic imaging), a history of vasovagal syncope, or hemodynamic instability.

### 2.4. Ethical Considerations

From the ethical point of view, all patients gave written consent for inclusion in the study. The process of the treatment was simplified and explained to the patients, once the physician assured that the patient completely understood the study protocol and became aware of his rights during the study, the written consent form was signed or fingerprinted by the patient. The institutional review board of Shahid Beheshti University of Medical Sciences approved the protocol of this study. The process of treatment had no harm for their health, and they had authority to stop the process of treatment.

In case of very rare incidence of side effects associated with PRP or autologous blood injection (persistent pain and swelling, infection and fibrosis, or any neuromuscular complications at injection site) patients had access to the project's physician in order to contact him if they encountered any of the possible adverse reactions to injection.

### 2.5. Randomization and Patients' Enrollment

The block covariate adaptive randomization method is designed to randomize subjects into the treatment groups. This led to equal sample sizes within each group and balance of the important covariates. Thus, a new participant is sequentially assigned to particular treatment groups by taking into account the specific matched covariates and previous assignments of participants.

### 2.6. Intervention

#### 2.6.1. Group 1 (Autologous PRP Group)

The treatment protocol for patients in this group was a single injection of 2 mL of autologous PRP, deep at the origin of wrist extensors, into maximal tenderness point at elbow region under aseptic technique.

Patients were referred to Shahid Modarres laboratory to extract and prepare PRP.

#### 2.6.2. PRP Preparation

The patient was placed in an appropriate and comfortable position that allows for sterility and access to the site of injection.

At first, 20 cc of venous blood was drawn with aseptic technique from venous antecubital vein and transferred to the centrifuge.

For the PRP preparation, the Rooyagen kit (made by Arya Mabna Tashkis Corporation, RN: 312569) approved by Iran Ministry of Health & Medical Education was used. For preparing 2 mL of PRP with concentration of 4–6 times the average normal values, 20 mL of blood was first collected from the patient's upper limb cubital vein using an 18G needle. Then 2 mL of ACD-A was added to the sample as an anticoagulant. One mL of the blood sample was sent for complete blood count. The rest of the sample passed two stages of centrifuge (first with 1600 rpm for 15 minutes for separation of erythrocytes and next with 2800 rpm for 7 minutes in order to concentrate platelets). The final product was 2 mL of PRP containing leukocytes. The PRP quantification and qualification procedure was performed using laboratory analyzer Sysmex KX 21 and if approved, the injection was proceeded.

#### 2.6.3. PRP Injection

The skin of the injection site was prepped and draped and the liquid PRP was injected in a sterile condition using a 22G needle at maximal tender point at elbow using a peppering technique spreading in a clock-like manner to achieve a more expansive zone of delivery.

#### 2.6.4. Group 2 (Autologous Whole Blood)

The patient is placed in an appropriate and comfortable position that allows for sterility and access to the site of injection.

Group 2 treatment protocol included a single injection of 2 mL of autologous peripheral whole blood under the same technique as the PRP group. Two mL of lidocaine 1% was injected 8 minutes before PRP or whole blood injection for patients in both groups.

Patients in both groups were observed in a supine position for 15–20 min afterwards to look for any adverse reaction to injection, then were discharged home.

No cortisone or nonsteroidal anti-inflammatories were prescribed during followup. For pain relief only, oral paracetamol and ice therapy were used. Patients of both groups were requested to refrain from heavy labor activities for a week. Tennis elbow strap (Oppo trademark) was administered for all patients and they were instructed to apply the strap 2 centimeters below the maximal tenderness point at elbow.

The patients were followed via weekly telephone calls and instructed how to use elbow splint and perform exercises. Three days after the injection, each patient was asked to start a simple program of extensor muscles stretching and 2 weeks after injection eccentric loading exercises were prescribed to be performed on an individual basis twice every day for 5 weeks. The patients were allowed to perform full activities of daily living after 4 weeks.

### 2.7. Outcome Measures

#### 2.7.1. Pain Intensity

Pain severity was evaluated before injection and reevaluation was done at 4 and 8 weeks, after the injection. Visual Analog Scale Analog Pain Score (VAS) (range, 0 [no pain] to 10 [agonizing pain]). The validity and reliability of self-rating scales like the VAS have previously been well described [13, 14]. Modified Mayo Clinic performance index score was used to evaluate functional outcome after the treatment.

### 2.8. Functional Outcome Measures

#### 2.8.1. Modified Mayo Clinic Performance Index

“Modified Mayo Clinic performance index” for the elbow was used as a valid and reliable measure to evaluate the functional improvement after therapy [15, 16]. The Mayo Clinic performance index for the elbow has 4 parameters: pain, motion, stability, and daily function. The maximum score is 100 and the minimum index is 0; the results are interpreted as excellent (≥90), good (75–89), fair (60–74), and poor (<60). The pain parameters in this questionnaire carries the highest points which is 45 out of 100 [16]. The modified mayo questionnaire was very specific to changes in elbow function. The questions were found to be reliable, reproducible and sensitive to change in elbow function [15]. Its construct validity is good for patient-rated variables and excellent for physician-rated variables. A minimal clinically important difference of 15 was reported for patients with rheumatoid arthritis after arthroplasty or synovectomy [17]. Mayo questionnaire was filled out via interviewing each patient before and after therapy.

#### 2.8.2. PPT

Pressure pain threshold (PPT) was assessed by algometer, Commander trademark. The PPT test is precise and reliable measurement for assessing pain (Cronbach's alpha ≥ 0.92). Pressure algometry has been shown to have good validity when assessed by pain and disability questionnaires (18). The algometer is comprised of a gauge attached to a hard rubber tip. Pressure was applied though the rubber surface area of 1 cm^2^ at a rate of 2 kg/cm^2^ per second. The instrument was placed perpendicular to the skin's surface. In each algometric assessment, we tested PPT at two different sites with 2 centimeters distance from each other at lateral epicondyle (site of maximal tenderness) and the mean of two values was considered as pain threshold. The method was demonstrated one time at each site before testing to ensure that the participants were familiar with the test. The participants were asked to indicate when the pressure became painful based on this definition: “When you feel the sensation changes from pressure to the slightest pain inform us.” Each measure site was tested three times with 2 minutes between each test, but the site was changed at each measure. The scale unit was kg/cm^2^.

### 2.9. Statistical Analysis

SPSS-16 (SPSS Inc Chicago, Illinois, United States of America) was used for data analysis. According to the Shapiro-Wilks normality tests, all variables had normal distribution so parametric tests including *t*-test, also Fisher's exact, GLM: repeated measure and Greenhouse-Geisser tests were run to compare these variables between two groups. *P* value less than 0.05 was considered significant. The assessors filling out the questionnaire and performing PPT, also the statistician, were blinded to the group of the patient.

## 3. Results

### 3.1. Patients' Characteristics

**Figure 1:**
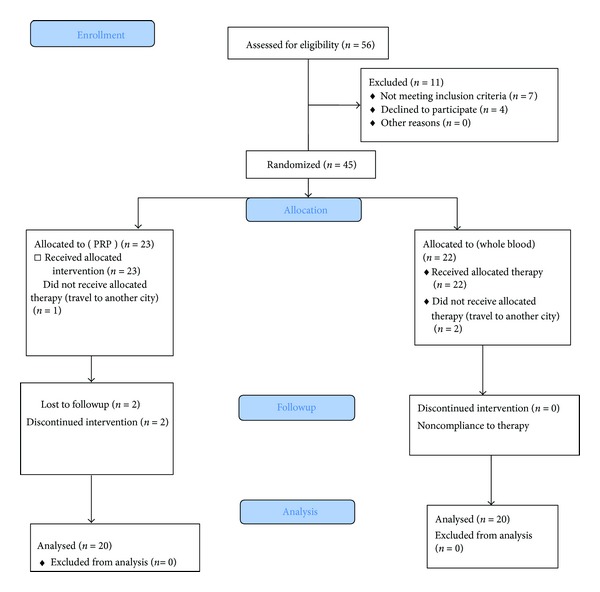
CONSORT 2010 flow diagram.

In this study, fifty-six patients were initially evaluated and 45 patients who had inclusion criteria entered the study and in the end 40 patients completed the study and their data was analyzed (twenty patients in each PRP and autologous group) (CONSORT flow chart) (Figure 1).

The mean age of patients was 46.25 ± 7.5 years old. Thirty-two patients were female (80%) and 8 patients were male (20%). All patients were right handed. The mean duration of symptoms in both groups was 14.5 ± 3 months. The patients' characteristics at study entry were shown in Table 1. There were no between-group differences at baseline in demographic characteristics and pain intensity at baseline (Table 1).

#### 3.1.1. PRP Characteristics

The mean platelets count of all patients at baseline was 220000/mm^3^ ± 23000, which increased to 990000 ± 43000 (4.5 times) in PRP preparation.

#### 3.1.2. Outcome Measures

All outcomes including VAS and Mayo scores and PPT were measured before intervention, then they were measured 4 and 8 weeks after initiating therapy in each group.

### 3.2. VAS Score

#### 3.2.1. Postintervention (4-Week Followup)

Mean VAS score decreased significantly in both PRP and AWB groups (*P* < 0.05).

#### 3.2.2. Postintervention (8-Week Followup)

Mean VAS score decreased significantly compared to 4 week only in PRP group (*P* < 0.05). VAS score did not change significantly compared to 4 week follow up at 8 week follow up in AWB group.

### 3.3. Mayo Score

#### 3.3.1. Postintervention (4-Week Followup)

Mayo score improved significantly in both PRP and AWB groups (*P* < 0.05).

#### 3.3.2. Postintervention (8-Week Followup)

Mayo score improved significantly compared to 4-week followup only in PRP group (*P* < 0.05). However, the change in Mayo score compared to 4-week followup was not significant in AWB group at 8-week followup (*P* > 0.05).

### 3.4. PPT Score

#### 3.4.1. Preintervention

PPT score was 17.8 ± 8.9 kg/cm^2^ (178 ± 89 N/cm^2^) (mean ± sd) in PRP group and 15.5 ± 5.2 kg/cm^2^ (155 ± 52 N/cm^2^) (mean ± sd) in AWB group.

#### 3.4.2. Postintervention (4-Week Followup)

Mean PPT score improved to 20 ± 5.9 kg/cm^2^ (200 ± 59 N/cm^2^) (mean ± sd) in PRP group and 19.7 ± 5.9 kg/cm^2^ (197 ± 59 N/cm^2^) (mean ± sd) in AWB group, which were statistically significant for both groups (*P* = 0.1, *P* = 0.09, resp.).

#### 3.4.3. Postintervention (8-Week Followup)

PPT scores did not improve significantly in both groups at 8-week followup (*P* > 0.05).

### 3.5. Between Group Comparisons

**Figure 2:**
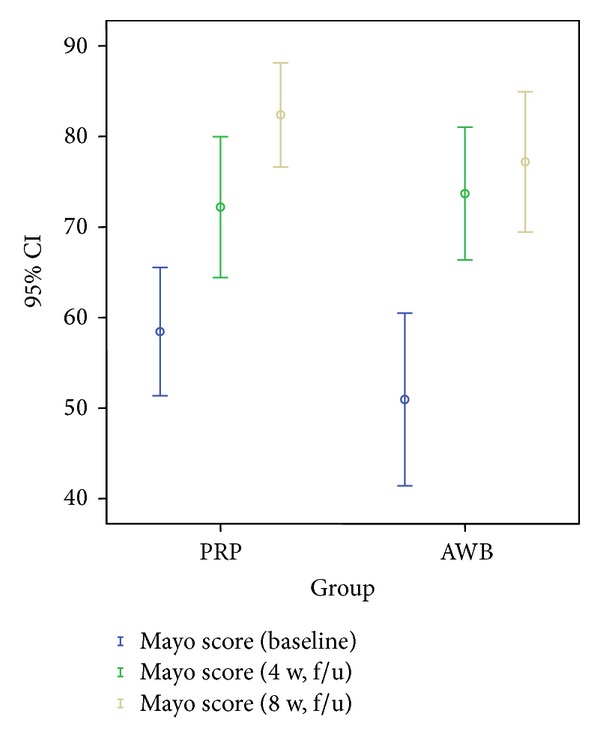
Mean of Mayo score in PRP and autologous whole blood (AWB) groups at baseline, 4 weeks, and 8 weeks after therapy.

**Figure 3:**
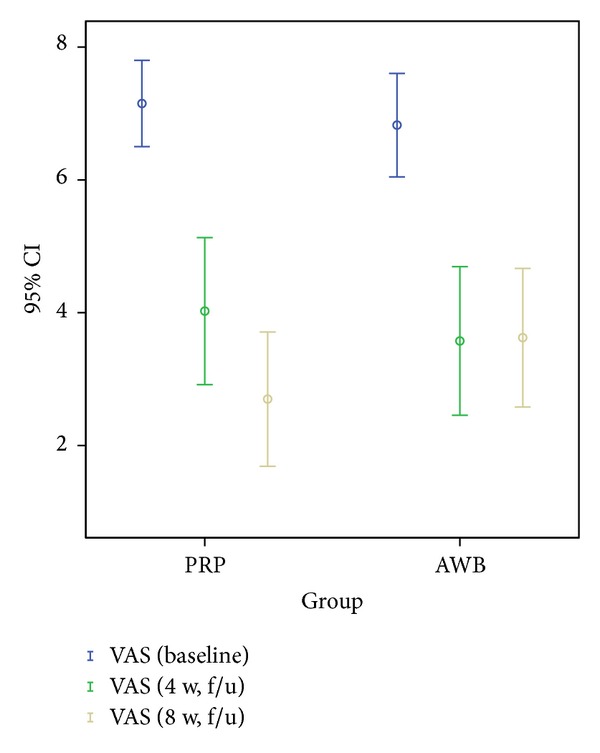
Mean of VAS at baseline in PRP and autologous whole blood (AWB) groups at baseline, 4 weeks, and 8 weeks after therapy.

**Figure 4:**
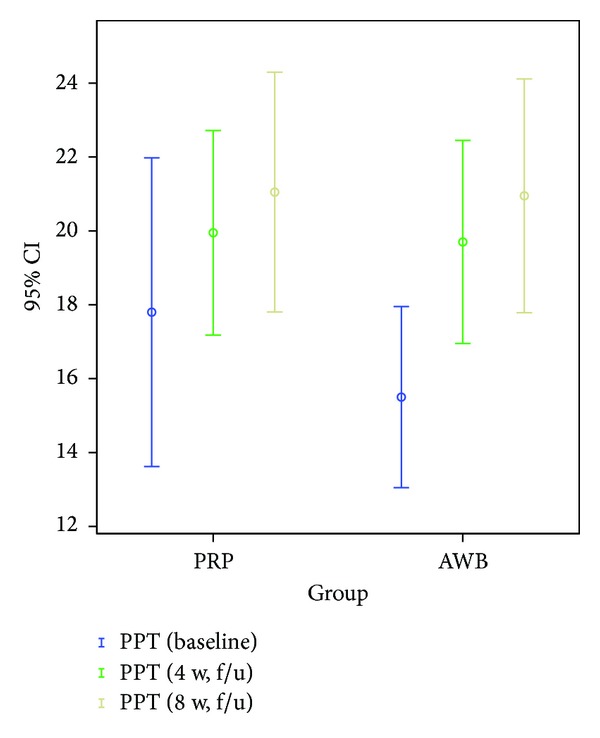
Mean of pain pressure threshold (PPT) in PRP and autologous whole blood (AWB) groups at baseline, 4 weeks, and 8 weeks after therapy.

No statistically significant difference was noted between two groups regarding pain scores in 4-week followup examinations (Table 2, Figures 2, 3, and 4).

However, at 8-week evaluations, pain improvement according to VAS and Mayo scores remained significant only in PRP group (Table 2, Figures 2, 3, and 4). PPT score did not improve significantly any further at 8-week followup compared to 4-week in both groups.

## 4. Discussion

According to the results of our study, local injection of PRP and autologous whole blood into lateral epicondyle both led to significant improvement in subjective (VAS) and objective pain scores (pain pressure threshold (PPT) measured by algometer) at 4-week follow-up examination in patients with lateral epicondylitis. Improvement in functional score was also noted according to Mayo score. There was no statistically significant difference between these two groups regarding pain and functional improvement in short-term followup. However, at 8-week follow-up examinations, this improvement in pain and functional status continued to be noted in VAS and Mayo scores only in PRP but not in control group. Mayo score improvement reached minimally clinically important difference reported for Mayo score change following therapy in inflammatory joint disease [17].

PPT score did not improve any further at 8-week followup compared to 4-week followup significantly in both groups.

In a study by Edwards and Calandruccio and Connell et al., the efficacy of autologous whole blood injection for pain relief in lateral epicondylitis was evaluated subjectively via Nirschl and VAS scale. Pain severity improved at the end of study, however, the mentioned studies lacked a control group [10, 11]. In 2006, Mirsha and his colleagues evaluated treatment of chronic severe elbow tendinosis with PRP. Eight weeks after the treatment, patients who had received PRP noted 60% improvement in their visual analog pain scores versus 16% improvement in control patients [3]. Pain and functional improvement were not evaluated objectively in above-mentioned studies. The strong point of our study compared to previous similar ones is that pain improvement was assessed via objective measures in addition to subjective scales.

In another double blind randomized clinical trial in 2010, the greater effect of PRP versus corticosteroids injection was shown. According to visual analog scores and DASH outcome measure scores (DASH: disabilities of the arm, shoulder, and hand), treatment of patients with chronic lateral epicondylitis with PRP reduced pain and significantly increased function more than corticosteroids [4].

Two RCTs were recently published in 2011 comparing autologous whole blood injection with PRP. In one of these RCTs. Thanasas evaluated the efficacy of PRP versus autologous blood in twenty-eight patients with tennis elbow. PRP and autologous groups received 3 mL of PRP and autologous whole blood, respectively. Evaluation using VAS and Liverpool elbow score was performed at 6 weeks, 3 months, and 6 months. Regarding pain reduction, PRP treatment seemed to be more effective and superior to autologous blood in the short term at 6 weeks [12] which is in agreement with the results of our study. However, in another study by Creaney et al., no differences were noticed in pain and disability up to six months after PRP or autologous blood injection in 150 patients, but there was a higher rate of conversion to surgery in the autologous blood group (20%) versus the PRP group (10%) [18].

The differences in sample size, 28 patients in Thanasas and 150 patients in Creaney may be a potential reason for differences between these two studies. The method of PRP preparations could be another source of different results obtained by these studies. As it was stated, therapeutic PRP should have a platelet concentration 4–6 times greater than whole blood and that concentrations lower than this may suppress healing. Hence, lower concentration of PRP preparations (2.8 times whole blood) in the study by Creaney could contribute to the lack of significant differences found in their study compared to Thanasas and our study [12, 18].

The effectiveness of PRP compared with corticosteroid injections in patients with chronic lateral epicondylitis was determined in a study by Peerbooms et al. They found that regarding pain reduction and functional improvement, corticosteroid was better initially and then declined, whereas the PRP group progressively improved; however, this study also lacked a control group [4].

In a systematic review published in 2008, Best et al. evaluated the results of five prospective case series and four controlled trials (three prolotherapy, two polidocanol, three autologous whole blood, and one platelet-rich plasma) for the treatment of refractory tennis elbow [19].

Three prospective case series assessing autologous whole blood reported significant (*P* < 0.05) improvement compared with baseline.

In a nonrandomised controlled trial [19] comparing a single treatment session of PRP with control injections, PRP subjects improved by a mean of 81% by 27 weeks. At 25.6 months, PRP patients further improved to 93% pain reduction compared with baseline.

Secondary outcome measures also improved in both PRP and whole blood groups. Mishra and Pavelko reported significant improvement on the Mayo Elbow-Performance Index after PRP therapy [3]. In the studies evaluated in this systematic review, whole blood injections reported significant improvement in functional scores and in maximal grip strength compared with baseline in the intervention groups.

They concluded that according to existing data for autologous whole blood and PRP injection, these therapies could be effective in treating tennis elbow, but as the authors concluded the results of this systematic review were limited by lack of large definitive clinical trials [19].

The exact mechanisms by which PRP initiates cellular and tissue changes are presently being investigated [20]. There is enough laboratory evidence of PRP effect on tendon healing and [21]. It has been considered in some studies that platelet growth factors could be effective in the cartilage healing process in knee osteoarthritis [22] PRP can stimulate processes associated with tendon healing. The proposed mechanism of action is the elicitation of a healing response in the damaged tendons by growth factors present in the blood [20]. These growth factors trigger stem cell recruitment, increase local vascularity, and directly stimulate the production of collagen by tendon sheath fibroblasts. Increased production of endogenous growth factors has been found in human tendons treated with PRP [3, 12, 21]. The above mechanism helps explain why a single PRP application can have a lasting effect on the healing process as it was shown in previous works of other authors investigating the long-term effect of PRP injection in chronic patellar or Achilles tendinopathy [23–25].

## 5. Conclusion

PRP and autologous whole blood injections are both effective methods to treat chronic lateral epicondylitis. However, at 8-week followup, PRP treatment seems to be a more effective treatment with more persistent efficacy than autologous blood in relieving pain and improving function.

Because PRP and whole blood are autologous and are prepared at the point of care, they have an excellent safety profile.

The limitation of our study was the relatively small number of cases included, absence of a control group receiving no intervention, and short-term follow-up evaluations. The second phase of this study is now being conducted to evaluate the long-term efficacy of PRP versus autologous blood at 8 months following injection. Another limitation of current study, also all similar studies mentioned above lack a control group; hence, whether these treatment approaches are superior to natural recovery remains unjustified.

We encourage more randomized clinical trials on this topic investigating the best technique of injection, number and time of injections, and number of platelets. Additionally, including control group who receive no therapy may let investigate the real efficacy of PRP compared to no treatment.

## Figures and Tables

**Table 1:** Demographic characteristics of patients in PRP and AWB groups.

Groups	(PRP)	(AWB)	*P* values
Sex			
Male	5 (25%)	3 (15%)	*P* = 0.7 Fisher exact test
Female	15 (75%)	17 (85%)	
Side of involvement			
Right	11 (55)	15 (75%)	*P* = 0.3 Fisher exact test
Side	9 (45%)	5 (25%)	
Age	47.2 ± 6.3	45.3 ± 8.7	*P* = 0.4 *t* test

**Table 2:** Mean of VAS and Mayo scores compared between three group at baseline (VAS0, MAYO0), at 4-week followup (VAS4, MAYO4) and at 8-week followup (VAS8, MAYO8). As it can be read from the table, at baseline there was no difference between two groups regarding these variables, at 4-week follow-up examinations, pain scores improved significantly in both groups but at 8-week followup, VAS and Mayo scores improved significantly only in PRP group.

Group	VAS0	VAS4	VAS8	MAYO0	MAYO4	MAYO8
PRP						
Mean ± SD	7.2 ± 1.4	4 ± 2.4	2.74 ± 2.2	58.42 ± 15.1	72.2 ± 16.6	82.4 ± 12.3
AWB						
Mean ± SD	6.8 ± 1.7	3.6 ± 2.4	3.6 ± 2.2	50.9 ± 20.4	73.7 ± 15.7	77.2 ± 16.5
*P* value	0.51	0.6	0.02	0.2	0.8	0.01
